# Prevalence and correlates of insufficient physical activity in school adolescents in Peru

**DOI:** 10.11606/S1518-8787.2018052000202

**Published:** 2018-05-03

**Authors:** Bimala Sharma, Rosemary Cosme Chavez, Eun Woo Nam

**Affiliations:** I Yonsei Global Health Center. Yonsei University. Wonju City, Republic of Korea; II Department of Health Administration. Graduate School. Yonsei University. Wonju City, Republic of Korea

**Keywords:** Adolescent Behavior, Physical Activity, Sedentary Lifestyle, Life Style, Risk Factors, Socioeconomic Factors, Health Surveys

## Abstract

**OBJECTIVE:**

To assess the prevalence and correlates of insufficient physical activity in adolescents in Peru.

**METHODS:**

We used a self-administered questionnaire developed from Global school-based Student Health Survey to collect information from secondary school students in North Lima and Callao in 2015. We carried out Poisson regression with robust variance using generalized linear models to estimate the crude and adjusted prevalence ratios (APR) with 95% confidence intervals (95%CI) of insufficient physical activity for its correlates.

**RESULTS:**

We have found that 78% of the adolescents did not meet the global recommendation of the World Health Organization on physical activity in the last week before the survey. Female respondents (APR = 1.13, 95%CI 1.04–1.21), respondents who perceived themselves as overweight (APR = 1.10, 95%CI 1.03–1.18), and respondents who consumed insufficient vegetables and fruits [no vegetables (APR = 1.30, 95%CI 1.06–1.59), no fruits (APR = 1.15, 95%CI 1.00–1.31) as compared to those who consumed ≥ 2 servings every day in the last seven days] were more likely to report insufficient physical activity. Adolescents who worked after school (APR = 0.92, 95%CI 0.84–0.99), had physical education classes five times per week (APR = 0.94, 95%CI 0.88–0.99), and had parental supervision (APR = 0.92, 95%CI 0.87–0.98) were less likely to report insufficient physical activity.

**CONCLUSIONS:**

Sex, work after school, perceived body weight, physical education class, parental support, and healthy dietary behaviors were associated with insufficient physical activity. Attempts to improve physical activity should look for ways to enhance leisure-time physical activity, parental support, physical education classes, healthy dietary behaviors, and normal body weight maintenance in adolescents with integrated efforts from the family and school.

## INTRODUCTION

Physical activity (PA) is any body movement produced by skeletal muscles that requires energy expenditure, including working, playing, doing household chores, traveling, and engaging in recreational pursuits. The World Health Organization (WHO) recommends that children and adolescents should have at least 60 minutes of moderate to vigorous intensity PA daily, which can be developmentally appropriate, enjoyable, and from a variety of activities^1^ ^,^ ^2^. However, approximately 80% of the adolescents worldwide do not meet the recommended daily level of PA, which means they are insufficiently physically active^2^ ^,^ ^3^. A study comparing 34 countries across five WHO regions has revealed that 23.8% of the boys and 15.4% of the girls met the recommendations of PA^4^. In Brazil, less than half of the adolescents reached the recommendation of PA, and this proportion tended to decrease among subjects with a higher socioeconomic level^5^. In the same vein, 24.5% of the students were physically active for a total of at least 60 minutes per day on five or more days during the past seven days in Peru, according to the Global school-based Student Health Survey (GSHS) conducted in 2010^6^.

Insufficient PA is a major risk factor for several non-communicable diseases^2^ ^,^ ^7^ ^,^ ^8^. A high level of PA during adolescence has been significantly associated with a high level of adult PA^9^ ^,^ ^10^. In addition, being overweight or obese as a child increases the risk of adult obesity^11^. Previous studies conducted in different countries show that insufficient PA is associated with several behavioral and socioenvironmental factors^12^ ^–^ ^18^.

Peru is an upper middle-income country experiencing steady economic growth since the last decade, with a life expectancy of 74.8 years and a human development index of 0.740^19^ ^,^ ^20^. Studies have shown a higher prevalence of overweight and obesity among children and adolescents in Lima compared to the rest of the country^21^ ^,^ ^22^. One of these studies has also shown that high socioeconomic status and living in Lima were associated with being overweight and obese^22^. Furthermore, it was also known that the lack of PA can have a greater impact on overweight and obesity than the amount of food consumed in school-age children in Lima^23^. This study tried to find out if the prevalence and correlates of insufficient physical activity in North Lima and Callao were similar with that in the rest of the world. We believe that the understanding of the correlates of insufficient PA may contribute with an effective planning for the promotion of PA. Therefore, this study aimed to assess the prevalence and correlates of insufficient physical activity among adolescents in North Lima and Callao, Peru.

## METHODS

### Study Design and Sampling

We conducted a cross-sectional survey among secondary school students in the Lima Metropolitan Area (Province of Lima and Callao), Peru. We used the information generated as part of the school health survey conducted by the Yonsei Global Health Center in collaboration with the Korea International Cooperation Agency, Peru Office. The survey was conducted in November 2015. The study participants were selected from four districts: Comas (Province of Lima), Bellavista, Ventanilla, and Mi Peru (Province of Callao) (Figure).

**Figure:**
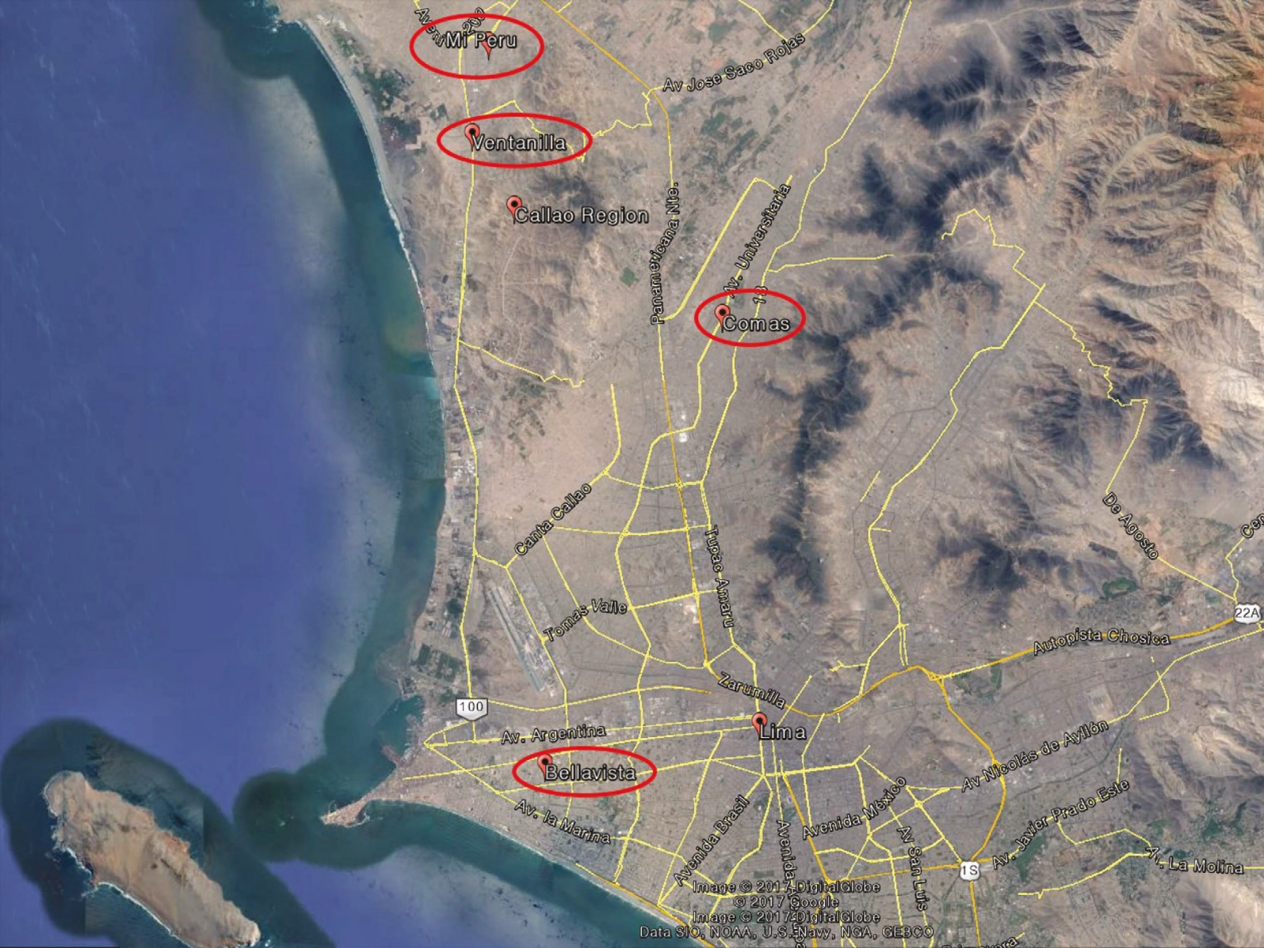
Map of the study areas.

The life expectancy of the study area was higher than the national average of 74.8 years, being it 78.9 years in Comas, 79.8 years in Bellavista, and 78.5 years in Ventanilla and Mi Perú^20^ ^,^ ^24^. The Mi Peru district has the largest population density, with over 24,000 inhabitants per square kilometer, followed by Bellavista district with almost 16,000 inhabitants. Comas has close to 11,000 inhabitants per square kilometer and Ventanilla has 5,017.93 inhabitants^25^ ^,^ ^26^. Three schools were selected from three areas of Comas: Santa Luzmila II, Laura Rodriguez Dulanto Duksil, and Carlos Phillips. Three schools were selected from the three districts in the Callao Province, one from each district. The study participants were secondary level school students from public schools. In Peru, a Ministerial Resolution has organized its basic education into three levels: initial, primary, and secondary. Secondary level education ranges from the first to the fifth grade and usually covers adolescents aged from 12 to 16 years^27^. All secondary classes were taken from each school and considered as strata. Students were chosen from each class using systematic random sampling with a random start. The sample size was calculated using the following formula as recommended by Naing et al. (2006), for the prevalence study^28^. A design effect (deff) of three was used to correct the sampling variance. Thus, we used the sample size = Z^2^ × P × (1 - P) / d^2^ × deff, where Z = Z statistic for a level of confidence (Z = 1.96), P = expected prevalence (p = 0.5), d = precision (d = 0.05), and deff = design effect (deff = 3). Therefore, the sample size calculated by the formula was 1,153. To minimize the effect of non-response and missing information, an additional 18% of the calculated sample was added as per the recommendation of 10% to 20% for the non-response rate^28^. Thus, we determined the total sample size of 1,360. From the data collected, 1,354 samples were selected for the analyses excluding the data with incomplete information.

### Measurement of Variables

#### Dependent variable

The following information was given to the participants before asking the particular question on PA: “You do PA by taking part in sports, playing with friends, or walking to school, riding a bike, dancing, playing soccer, playing volleyball, etc.”, and “During the last seven days, on how many days did you do any type of PA for a total of at least 60 minutes a day?”^29^. Physical activity was measured using this single self-reported question, as it has been used in some previous studies^13^ ^,^ ^15^ ^,^ ^30^. The options given to answer were from zero to seven days. Based on the WHO recommendation of at least 60 minutes of PA each day, adolescents were classified into two groups: those who met the recommendation for all days in the last week preceding the survey, grouped as sufficiently physically active, and those who did not comply with this standard, grouped as inactive or insufficiently physically active group.

#### Independent variables

For smoking and alcohol consumption, current users were those who had smoked or drank alcohol within the last 30 days before the survey, and former smokers or former drinkers were those who had smoked or drank alcohol at least once in their lives. The option “do not remember” was treated as a missing value. “In the last seven days, how many times did you eat vegetables?” was asked to find out the vegetable consumption of the participants. The options were: none in the last seven days, once or twice a week, three to four times a week, five to six times a week, once a day, and at least twice a day. To assess fruit consumption, participants were asked the following question: “In the last seven days, how many times did you eat fruit?” The options were the same as those for vegetable consumption. We asked “Are you participating in any activity after school that helps you to earn money for your family, personal expenses, or any other end?” to measure whether the participants worked after school. To determine the degree of parental supervision of the adolescents, we asked them how often their parents or guardians checked their homework during the past 30 days. Participants could choose from always, most of the time, sometimes, rarely, and never. The question “How often did you spend time with your parents or guardians during the past 30 days?” was asked to assess the time spent with parents, and the options were similar to that of parental supervision. We also asked “How many days a week do you attend a physical education class at school?”, and the responses were categorized as either less than five or five or more regular physical education classes. Moreover, we asked them “What is your family's economic status?”, and adolescents could choose between the high, above average, average, below average, and low options. For the question of how they would rate their health in general, responses were dichotomized into excellent, very good, or good, and fair or poor. For perceived body weight, we asked “How do you perceive your body weight?” The options of very underweight or underweight were grouped into the underweight group, slightly overweight, overweight, or very overweight were grouped into the overweight group, and normal weight was used as the reference category.

### Information Collection

A self-administered standard questionnaire was provided to the selected students in their respective classrooms. Ten trained enumerators gave a brief orientation on the objectives of the study and ways to fill the questionnaire before collecting the information. As a tool to collect information, the GSHS Questionnaire was slightly modified and translated into Spanish. The frequencies of fruit and vegetable intake were also modified in terms of the recall period of seven days instead of the 30 days in the original questionnaire^16^. The GSHS Questionnaire is a self-administered questionnaire to assess behavioral risk factors and protective factors in adolescents^29^. This survey was voluntary and anonymous. Teachers and school staff were not allowed to attend the students while filling the questionnaire. We did not provide any incentive for subjects to participate in the survey.

### Statistical Analysis

We analyzed the data collected using the Statistical Package for the Social Sciences (SPSS) version 21 for Windows (IBM Corp.: Armonk, NY, USA). We carried out Pearson chi-square test between each independent variable and insufficient PA. The level of significance was set at 5%. All significant variables from the chi-square test were selected for the multivariable analyses. Poisson regression with robust variance was performed using generalized linear models to estimate prevalence ratios (PR) of insufficient PA as per the recommendation provided by Barros and Hirakata^31^. School was adjusted as a categorical variable in the multivariable analysis. We performed crude and adjusted prevalence ratios (APR) with 95% confidence intervals (CI). In addition, we found no effect of multicollinearity between the independent variables (variance inflation factor < 3).

### Ethical Approval

We obtained ethical approval for the study from the Institutional Review Board of the Wonju Campus of Yonsei University (IRB 1041849-201510-BM-092-03) and from the *Dirección Regional de Salud* (DIRESA Callao, Peru). Consent was also given in advance by each school administration, as well as the parents or guardians of the participants. There were no refusals to participate in the study. Informed consent was provided by the individual students before they filled out the questionnaire.

## RESULTS

Most participants were female (61.5%) and were in the age group of 11 to 14 years (55.2%). Of the total respondents, 61.8% reported their economic status as average, and 23.5% reported they worked after school. Approximately half of the students (49.8%) reported physical education classes every day in the last week. Among them, 60% mentioned that their parents always or most of the time checked their homework, and 49.6% reported that their parents always or most of the time spent time with them during the past 30 days. Of the total, 31.2% reported fair or poor self-rated health and 30.6% perceived themselves as overweight, while 9.3% and 19.1% reported they smoked and drank alcohol, respectively, in the last 30 days preceding the survey. Only 16.8% and 6.5% of the respondents consumed two or more than two servings of fruits and vegetables per day in the last 30 days, respectively. Of the total, 78.1% of the adolescents did not meet the recommended level of PA each day for all days in the last week before the survey, and 11.2% did not meet the standard even for a single day within the studied week. The average number of days that adolescents followed the recommendation of PA in the week was 3.4 days (Table 1).

**Table 1:** General characteristics of the study population and prevalence of insufficient physical activity. (n = 1,354)

Variable		n	Percent/Mean (SD)
Sex			
	Male	521	38.5
	Female	833	61.5
Age group (in years)			
	11–14	747	55.2
	15–19	607	44.8
Perceived economic status			
	High/Above average	363	26.8
	Average	837	61.8
	Below average and low	148	10.9
Work after school			
	Yes	318	23.5
	No	1,032	76.2
	Missing	4	0.3
Physical education class per week			
	5 days	674	49.8
	< 5 days	670	49.5
	Missing	10	0.7
Parental supervision			
	Most of the time/Always	813	60.0
	Never/Rarely/Sometimes	536	39.6
	Missing	5	0.4
Spending time with parents			
	Most of the time/Always	672	49.6
	Never/Rarely/Sometimes	673	49.7
	Missing	9	0.7
Self-rated health			
	Excellent/Very good/Good	927	68.5
	Fair/Poor	423	31.2
	Missing	4	0.3
Perceived body weight			
	Underweight	272	20.1
	Normal	664	49.0
	Overweight	415	30.6
	Missing	9	0.7
Smoking			
	Current	126	9.3
	Former	190	14.0
	Never	978	72.2
	Missing	60	4.4
Alcohol consumption			
	Current	258	19.1
	Former	241	17.8
	Never	766	56.6
	Missing	89	6.6
Fruit consumption			
	None	104	7.7
	1–6 times a week	890	65.7
	Every day	125	9.2
	≥ 2 servings each day	228	16.8
	Missing	7	0.5
Vegetable consumption			
	None	287	21.2
	1–6 times a week	836	61.7
	Every day	140	10.3
	≥ 2 servings each day	88	6.5
	Missing	3	0.2
Insufficient physical activity*			
	No	297	21.9
	Yes	1,057	78.1
Number of days with at least 60 minutes of physical activity			
	Mean days	1,354	3.42 (2.4)
Number of days with at least 60 minutes of physical activity			
	None	125	11.2

* 68.1% of male and 84.3% of female.

Table 2 shows the results of the chi-square test between the independent variables and insufficient PA. Work after school, physical education class, parental supervision, and spending time with their parents, as well as self-rated health, perceived body weight, alcohol consumption, smoking, and vegetable and fruit consumption had significant associations with insufficient PA.

**Table 2:** Association of the independent variables with insufficient physical activity in adolescents.

Variable		Insufficient physical activity		p
		Yes	No	
		n (%)	n (%)	
Age (in years)				
	11–14	585 (78.3)	162 (21.7)	0.807
	15–19	472 (77.8)	135 (22.2)	
Sex				
	Female	702 (84.3)	131 (15.7)	< 0.001
	Male	355 (68.1)	166 (31.9)	
Work after school				
	Yes	226 (71.1)	92 (28.9)	0.001
	No	827 (80.1)	205 (19.9)	
Perceived economic status				
	High/Above average	272 (74.9)	91 (25.1)	0.202
	Average	666 (79.6)	171 (20.4)	
	Below average and low	115 (77.7)	33 (22.3)	
Physical education class per week				
	< 5 days	548 (81.8)	122 (18.2)	0.001
	5 days	500 (74.2)	174 (25.8)	
Parental supervision				
	Most of the time/Always	617 (75.9)	196 (24.1)	0.018
	Never/Rarely/Sometimes	436 (81.3)	100 (18.7)	
Spending time with parents				
	Most of the time/always	505 (75.1)	167 (24.9)	0.010
	Never/Rarely/Sometimes	545 (81.0)	128 (19.0)	
Self-rated health				
	Excellent/Very good/Good	707 (76.3)	220 (23.7)	0.018
	Fair/Poor	347 (82.0)	76 (18.0)	
Perceived body weight				
	Underweight	207 (76.1)	65 (23.9)	< 0.001
	Normal	497 (74.8)	167 (25.2)	
	Overweight	352 (84.8)	63 (15.2)	
Alcohol consumption				
	Current	185 (71.7)	73 (28.3)	0.021
	Former	195 (80.9)	46 (19.1)	
	Never	606 (79.1)	160 (20.9)	
Smoking				
	Current	87 (69.0)	39 (31.0)	0.001
	Former	138 (72.6)	52 (27.4)	
	Never	790 (80.8)	188 (19.2)	
Vegetable consumption				
	None	238 (82.9)	49 (17.1)	< 0.001
	1–6 times a week	662 (79.2)	174 (20.8)	
	Every day	100 (71.4)	40 (28.6)	
	≥ 2 servings each day	55 (62.5)	33 (37.5)	
Fruit consumption				
	None	86 (82.7)	18 (17.3)	< 0.001
	1–6 times a week	717 (80.6)	173 (19.4)	
	Every day	97 (77.6)	28 (22.4)	
	≥ 2 servings each day	152 (66.7)	76 (33.3)	

We computed the crude and adjusted prevalence ratios of insufficient PA for the explanatory variables in Table 3. According to this multivariable analysis, female adolescents were 13% more likely to report insufficient PA. Adjusted prevalence ratio (APR) of insufficient PA was 0.92 among adolescents who were involved with work after school, which indicates lower probability of insufficient PA among them. Adolescents who attended five physical education classes each week were also less likely to report insufficient PA than those who mentioned less than five physical education classes. In the same way, APR was less than one among adolescents whose parents checked their homework. Adolescents who perceived themselves as being overweight were more likely to report insufficient PA as compared to those who perceived themselves as having a normal weight. We found no significant association between alcohol consumption or smoking and insufficient PA in the adjusted model. Adolescents who did not consume vegetables and those who consumed them 1–6 times were 30% and 25%, respectively, more likely to report insufficient PA compared to those who consumed two or more servings in a day. In addition, adolescents who did not eat fruits and those who ate fruits only 1–6 times a week were more likely to do insufficient PA, compared to those who ate two or more servings of fruit each day [no fruits (APR = 1.15, 95%CI 1.00–1.31) and consumption 1–6 times a week (APR = 1.14, 95%CI 1.03–1. 27)].

**Table 3:** Crude and adjusted prevalence ratios (PR) of insufficient physical activity for explanatory variables in adolescents.

Variable		Crude PR	Adjusted^a^ PR
		(95%CI)	(95%CI)
Sex			
	Female	1.23 (1.15–1.32)^b^	1.13 (1.04–1.21)^c^
	Male	1	1
	Age	1.00 (0.98–1.02)	1.01 (0.99–1.04)
Work after school			
	Yes	0.88 (0.82–0.95)^c^	0.92 (0.84–0.99)^d^
	No	1	1
Physical education class per week			
	5 days	0.90 (0.85–0.96)^c^	0.94 (0.88–0.99)^d^
	< 5 days	1	1
Parental supervision			
	Most of the time/Always	0.93 (0.88–0.98)^d^	0.92 (0.87–0.98)^d^
	Never/Rarely/Sometimes	1	1
Spending time with parents			
	Most of the time/Always	0.92 (0.87–0.98)^d^	0.95 (0.90–1.01)
	Never/Rarely/Sometimes	1	1
Self-rated health			
	Excellent/Very good/Good	0.93 (0.87–0.98)^d^	0.99 (0.93–1.06)
	Fair/Poor	1	1
Perceived body weight			
	Underweight	1.01 (0.93–1.10)	1.02 (0.94–1.10)
	Overweight	1.13 (1.06–1.20)^b^	1.10 (1.03–1.18)^c^
	Normal	1	1
Alcohol consumption			
	Current	0.90 (0.83–0.98)^d^	0.91 (0.82–1.00)
	Former	1.02 (0.95–1.09)	1.01 (0.93–1.09)
	Never	1	1
Smoking			
	Current	0.85 (0.75–0.96)^d^	0.93 (0.82–1.05)
	Former	0.89 (0.82–0.98)^d^	0.93 (0.84–1.02)
Never			
Vegetable consumption			
	None	1.32 (1.11–1.57)^c^	1.30 (1.06–1.59)^c^
	1–6 times a week	1.26 (1.07–1.49)^c^	1.25 (1.02–1.52)^d^
	Every day	1.14 (0.94–1.38)	1.24 (1.00–1.55)^d^
	≥ 2 servings a day	1	1
Fruit consumption			
	None	1.24 (1.09–1.40)^c^	1.15 (1.00–1.31)^d^
	1–6 times a week	1.20 (1.09–1.33)^b^	1.14 (1.03–1.27)^c^
	Every day	1.16 (1.02–1.32)^d^	1.09 (0.95–1.24)
	≥ 2 servings a day	1	1

a The variables entered into the adjusted analysis were sex, age (continuous variable), work after school, physical education class per week, parental supervision, spending time with parents, self-rated health, perceived body weight, alcohol consumption, smoking, vegetable consumption, fruit consumption, and school.

b p < 0.001

c p < 0.01

d p < 0.05

## DISCUSSION

Nearly four-fifths (78%) of the surveyed adolescents did not meet the global recommendations for PA for the last week before the survey, which is similar to the global prevalence^2^ ^,^ ^3^. Female adolescents were more likely to report insufficient PA in the study, and this finding is consistent with most other studies conducted in different countries^16^ ^,^ ^17^ ^,^ ^32^ ^–^ ^34^. Most of the studies on PA have considered at least 60 minutes of PA for all days in the last seven days; however, some studies have classified at least 60 minutes of PA on five or more days each week as an active level of exercise^5^. Although many studies indicate that PA decreases with an increase in age, we observed no association between the age of the participants and insufficient PA in the study, which may reflect that they are inactive from an early adolescent age^11^ ^,^ ^32^ ^,^ ^35^. The behavior of PA may go from adolescence to adulthood. Participants who engage in regular PA during adolescence are more likely to be adequately active in adulthood. Therefore, the promotion of PA while persons are still of school age may be a successful intervention against the epidemic of adult inactivity^9^.

A review study has stated that adolescents with higher socioeconomic status are more physically active than those with a lower socioeconomic status^36^. Our study found that adolescents who worked after school to make money for their family, personal expenses, or any other end were less likely to report being physically inactive. However, this does not mean asking parents to involve their children in paid work after school. Instead, efforts to improve PA should search ways to increase leisure-time PA. There was no association between self-reported socioeconomic status and PA in this study.

Adolescents who took part in regular physical education classes had lower odds of not following the recommendation of PA. This finding is consistent with other studies in different countries^14^ ^,^ ^17^. Interventions in schools have the potential to change the PA levels of young persons, and other school-based strategies that encompass physical education and afterschool sports are some of the evidence-based interventions to tackle insufficient PA^11^ ^,^ ^37^. Hoehner et al. (2008) have also recommended that the implementation of school physical education programs should be strongly encouraged to promote the health of Latin American children^38^. In Peru, a Ministerial Resolution establishes Physical Education as a required class for the Primary and Secondary level, with two pedagogical sessions of 45 minutes per week^27^. As in our study, adolescents whose parents almost always checked their homework were less likely to be physically inactive than those who had less involved parents among South East Asian adolescents^30^. Systematic review studies have therefore concluded that parental support has an influence on the PA level of adolescents^11^ ^,^ ^17^.

In our study, we found a significant relation between PA and self-rated health in the chi-square analysis; however, insufficient PA was not significantly associated with self-rated health in the multivariable analysis. A previous study found that physical activity of at least 1 hour each day for three or more days per week was associated with a reduced likelihood of poor or fair self-rated health. The difference might be due to the cut-off value of sufficient PA; at least one hour of PA for ≥ 3 days was measured as an active lifestyle in the aforementioned study^39^. A significant association of low self-rated health has also been found by a follow-up study with boys active for < 4 hours per week compared with those active for > 4 hours per week among Swedish adolescents^40^. Thus, the measurement of PA and other covariates might influence the association between PA and self-rated health.

In this study, participants who perceived themselves as overweight had higher odds of being physically inactive compared to those who perceived themselves as normal. This finding is consistent with a study that has stated that German adolescents who judged themselves as “too fat” were affected by a higher risk of less frequent PA^41^. Similarly, persons who perceive themselves as underweight were also slightly more likely to be inactive as compared to a person of normal weight^42^. Body image rather than body mass index is an important factor for PA in adolescents and should be considered in the formulation of programs aimed at improving PA in this age group^43^.

The General Law for the Prevention and Control of the Risks from Tobacco Consumption and the Law that regulates the commercialization, consumption, and advertising of alcoholic beverages established, in 2006, 18 as the legal age for smoking and consumption of alcoholic beverages in Peru^44^ ^,^ ^45^. Nevertheless, a large proportion of adolescents smokes and drinks. However, we observed no association between PA and alcohol consumption and smoking in the study. Smoking has been found to be associated with PA level among Taiwanese adolescents^30^. A review study has suggested that adolescents who participate in sports reported higher levels of alcohol consumption but lower levels of cigarette smoking^46^.

Insufficient PA and an unhealthy diet are the leading risk factors for non-communicable diseases^7^. In a study, adolescents consuming inadequate fruits and vegetables were more likely to report being physically inactive; in other words, the study has found that the most active adolescents consumed fruit and vegetables more frequently than their less-active peers^34^. A study with Brazilian adolescents has also revealed that those who consumed fewer servings of fruit and vegetable a day had a greater chance of being inactive^15^. Another study with Southeast Asian adolescents has found that those who ate less than three servings of vegetables each week were more likely to not follow the recommendations of PA^30^. However, we cannot conclude that an improved healthy diet will improve PA. Instead, overall healthy habits are associated with each other.

Based on the multivariable Poisson regression analysis, sex, physical education class, parental support, perceived body weight, and fruit and vegetable consumption as correlates were consistent with other studies, whereas age, work after school, self-reported socioeconomic status, smoking, and alcohol consumption were different findings in our study.

This study has some limitations. First, as the study is based on a cross-sectional assessment, we cannot draw causal inferences for explanatory variables based on our results. Second, there could be other correlates of PA that we did not assess in the study. This study did not include variables on enabling environments for PA at home and in the community. Third, as all the measurements were self-reported, there is room for possible reporting bias, in which the participants may underreport or over report their behaviors. Fourth, the question about PA used did not specifically include items about household chores, and it did not provide any instructions specifically about the treatment of physical activity during the physical education class. Thus, there might be some room for underreporting of the status of PA.

## CONCLUSIONS

Approximately 80% of the adolescents did not meet the recommendation of PA in the last week before the survey. Female respondents, respondents who perceived themselves as overweight, and respondents who consumed an inadequate amount of vegetables and fruits were more likely to report insufficient PA, whereas respondents who worked after school, had more physical education classes, and had parental supervision were less likely to do insufficient PA. Efforts to improve PA in adolescents should try to enhance leisure-time PA, parental support, physical education classes, healthy dietary behaviors, and normal body weight maintenance. Healthy dietary habits are significantly correlated with PA, which may be because overall healthy habits are associated with each other; thus, this supports the need for an integrated approach for health promotion in adolescents. A joint effort of the family and school may contribute to promote PA among children and adolescents.
